# Netrin-1 Protects against L-Arginine-Induced Acute Pancreatitis in Mice

**DOI:** 10.1371/journal.pone.0046201

**Published:** 2012-09-27

**Authors:** Ji Chen, Qing-ping Cai, Pi-jie Shen, Rong-lin Yan, Chang-ming Wang, De-jun Yang, Hong-bing Fu, Xue-yun Chen

**Affiliations:** Department of Gastrointestinal Surgery, Shanghai Changzheng Hospital, Second Military Medical University, Shanghai, China; University of Valencia, Spain

## Abstract

Acute pancreatitis (AP) is a common inflammatory disease mediated by damage to acinar cells and subsequent pancreatic inflammation with infiltration of leukocytes. The neuronal guidance protein, netrin-1, has been shown to control leukocyte trafficking and modulate inflammatory responses in several inflammation-based diseases. The present study was aimed toward investigating the effects of netrin-1 in an *in vivo* model of AP in mice. AP was induced in C57BL/6 mice by administration of two intraperitoneal injections of L-Arginine (4 g/kg). Mice were treated with recombinant mouse netrin-1 at a dose of 1 µg/mouse or vehicle (0.1% BSA) intravenously through the tail vein immediately after the second injection of L-Arginine, and every 24 h thereafter. Mice were sacrificed at several time intervals from 0 to 96 h after the induction of pancreatitis. Blood and tissue samples of pancreas and lung were collected and processed to determine the severity of pancreatitis biochemically and histologically. Immunohistochemical staining demonstrated that netrin-1 was mainly expressed in the islet cells of the normal pancreas and the AP model pancreas, and the pancreatic expression of netrin-1 was down-regulated at both the mRNA and protein levels during the course of AP. Exogenous netrin-1 administration significantly reduced plasma amylase levels, myeloperoxidase activity, pro-inflammatory cytokine production, and pancreas and lung tissue damages. Furthermore, netrin-1 administration did not cause significant inhibition of nuclear factor-kappa B activation in the pancreas of L-Arginine-induced AP. In conclusion, our novel data suggest that netrin-1 is capable of improving damage of pancreas and lung, and exerting anti-inflammatory effects in mice with severe acute pancreatitis. Thus, our results indicate that netrin-1 may constitute a novel target in the management of AP.

## Introduction

Acute pancreatitis (AP) is an inflammatory condition with a clinical course that varies from mild to severe [1]. Severe AP remains a life-threatening disease with a high mortality rate among a defined proportion of those affected [2]. The current management of AP is limited to supportive care and treatment of complications when they develop; thus, an effective treatment is urgently needed [3], [4]. In past decades, intense efforts have been devoted to elucidating the events responsible for the initiation and severity of the disease so that novel therapeutic targets may be identified. Although the exact mechanisms are for the most part unknown, there is some evidence that the severity and outcome of AP might be determined by the acinar cell response to activation of trypsinogen, as well as the events that occur subsequent to acinar cell injury, including activation of transcription factors such as nuclear factor-kappa B (NF-κB), recruitment of inflammatory cells, and generation of inflammatory mediators [1], [5]. In light of this knowledge, inhibition of the inflammatory pathway seems to be the most promising approach for preventing the development of the disease.

Over the past few years, the neuronal guidance protein, netrin-1, has received considerable attention for its potential role in inflammation-based diseases. Netrin-1 was originally identified as a diffusible factor released by neural floor plate cells in the developing spinal cord that regulates axonal outgrowth and growth cone migration [6], [7]. Subsequent studies found that netrin-1 is also expressed outside the central nervous system and controls leukocyte trafficking from the vascular space into sites of acute inflammation [8], [9]. Recent studies in various inflammation-based diseases, such as kidney ischemia reperfusion injury, hypoxia-induced inflammation, acute lung injury, peritonitis, and inflammatory bowel disease, showed that netrin-1 holds anti-inflammatory potential and can reduce local inflammatory tissue injury [10]–[14]. However, the role of netrin-1 in acute pancreatitis, to the best of our knowledge, has not been investigated to date.

In the developing pancreas, netrin-1 acts as a regulator of cell adhesion, migration, and differentiation [15]. It was thought that netrin-1 was absent in the normal adult pancreas, but netrin-1 has been found in pancreatic adenocarcinoma and is implicated in tumorigenesis [15], [16]. In addition, a recent study revealed that netrin proteins are present in adult pancreatic islets, and netrin-1 modulates beta cell apoptosis signaling via dependence receptors [17]. These findings suggest that netrin-1 may be involved in the progression of pancreatic diseases. AP is a common inflammatory disease mediated by damage to acinar cells and subsequent pancreatic inflammation with recruitment of leukocytes [1], [5]. We first hypothesized that netrin-1 could protect against acute pancreatitis by inhibiting leukocyte infiltration and suppressing the inflammatory response. To test this hypothesis, we used recombinant mouse netrin-1 to investigate the effects of netrin-1 during experimental pancreatitis. We found that exogenous netrin-1 administration could reduce plasma amylase levels, myeloperoxidase (MPO) activity, pro-inflammatory cytokine production, and pancreatic and pulmonary damage in the mouse model of L-Arginine-induced AP.

## Materials and Methods

### Experimental procedures

All animal experiments were approved by the Animal Research Committee at Shanghai Second Military Medical University and were carried out in accordance with established International Guiding Principles for Animal Research. C57BL/6 mice (male, 20–25 g) were used and maintained in the Animal Housing Unit in an environment with controlled temperature (21–24°C) and lighting (12 h light-dark cycle). Standard laboratory chow and drinking water were provided *ad libitum*. A period of at least 5 days was allowed for the animals to acclimatize before any experimental procedures were undertaken.

### Induction of AP

L-Arginine hydrochloride was purchased from Sigma Chemical (Sigma-Aldrich, St. Louis, MO, USA). Mice were randomly assigned to three groups. Group 1: Animals were given two intraperitoneal injections of normal saline, 1 h apart (Normal group). Group 2: Animals were injected intraperitoneally with L-Arginine in two dose of 4 g/kg body weight each, 1 h apart (AP group) [18]. Group 3: Mice were administered recombinant mouse netrin-1 (R&D Systems, Minneapolis, MN) at a dose of 1 µg/mouse intravenously through the tail vein in a volume of 0.1 ml immediately after the second injection of L-Arginine (0 h) and every 24 h thereafter (AP+Netrin-1 group). The normal group and AP group of mice received similar injections of vehicle (0.1% BSA) at the same time point. Animals were sacrificed at several time intervals from 0 to 96 h after induction of AP. Blood samples were taken to determine serum amylase and cytokine levels. The pancreas and right lung were rapidly removed from each mouse for morphologic examination and scoring. Portions of these organs were also stored at −80°C for further investigation.

### Morphological examination

Samples of pancreas and lung were fixed in 4% paraformaldehyde buffered with PBS (pH 7.4). The tissues were then embedded in paraffin, and 5 µm sections were processed for hematoxylin and eosin (H&E) staining by standard procedures. The pancreatic damage was evaluated in a blinded manner using a preexisting scoring system on a 0 (absent) to 4 (extensive) scale, as previously described [19]. Total histological scores of pancreas were obtained, representing the sum of the scores for edema, inflammatory cell infiltration, hemorrhage, and acinar cell necrosis. The lung injury was scored according to inflammatory changes, pulmonary edema, and hemorrhage of alveoli and interstitial tissue. Each pathological change was scored on a scale from 0–3 (normal, 0; minimal change, 1; medium change, 2; and severe change, 3).

### Amylase estimation

Plasma amylase activity was measured using a kinetic spectrophotometric assay. Plasma samples were incubated with amylase reagent (Sigma) for 2 min at 37°C, and absorbance was measured every minute for the subsequent 2 min at 405 nm according to the manufacturer's instructions. The resulting change in absorbance was used to calculate amylase activity.

### MPO estimation

Neutrophil sequestration in pancreas and lung was quantified by measuring tissue MPO activity. For the measurements, tissue samples were immediately homogenized on ice in 5 volumes of normal saline. MPO activity was measured using the MPO assay kit (Nanjing Jiancheng Corp., Nanjing, China), following the manufacturer's recommendations. One unit of MPO activity is defined as degrading 1 µmol of hydrogen peroxide at 37°C; MPO activity of tissue was expressed as unit per gram (U/g).

### Measurement of plasma cytokines

The plasma levels of cytokines (TNF-α, IL-1β, IL-6, and IL-10) were measured by the enzyme-linked immunosorbent assay (ELISA) in accordance with the manufacturer's manual using an ELISA kit (R&D Systems, Minneapolis, MN) for mouse TNF-α , IL-1β, IL-6, and IL-10. Concentrations were expressed as picograms per milliliter (pg/ml).

### Reverse transcription and quantitative real-time PCR

Total cellular RNA was extracted from 50 to 100 mg tissue using Trizol reagent (Invitrogen, Carlsbad, CA) according to the manufacturer's protocol. RNA concentration and purity were measured by NanoDrop 2000 (Thermo Scientific, Waltham, MA, USA). First-strand complementary DNA was synthesized with M-MLV reverse transcriptase (Promega, Madison, WI, USA) using Oligo-dT as a primer. Equal amounts of RNA (2 µg) were used as templates in each reaction. Quantitative real-time PCR was performed with SYBR Green PCR master mix (Applied Biosystems, Foster City, CA, USA) on the Applied Biosystems 7900HT system using primers for Netrin-1, NF-κB (p65), and β-actin (Netrin-1-F: AAGCCTATCACCCACCGGAAG; Netrin-1-R: GCGCCACAGGAATCTTGATGC; NF-κB -F: ATACCACCAAGACCCACCCC; NF-κB -R: TGAGGAGGGTCCTTGGTGAC; β-actin-F: AGAGGGAAATCGTGCGTGAC; β-actin-R:CAATAGTGATGACCTGGCCGT). All experiments were repeated at least three times, and the comparative CT (ΔΔCt) method was used to calculate relative fold changes in gene expression using β-actin mRNA expression levels for normalization.

### Western blot analysis

The pancreatic tissues were homogenized in RIPA lysis buffer. Aliquots from tissue lysate (about 20 µg protein) were separated by sodium dodecyl sulfate–polyacrylamide gel electrophoresis (SDS–PAGE) and blotted onto polyvinylidene difluoride membranes (Millipore Corp, Bedford, MA, USA). Membranes were blocked in 5% milk, and then probed with antibodies against Netrin-1 (1∶1000, abcam, Cambridge, UK) and NF-κB (1∶1000, abcam) at 4°C overnight. After being washed, the membranes were incubated with horseradish peroxidase-conjugated secondary antibodies (Cell Signaling, Beverly, MA, USA) and visualized by diaminobenzidine (DAB) staining. The band concentration was quantified with the BIO-RAD imaging system. β-actin (1∶1000, abcam) was used as a loading control.

### Immunohistochemical staining of Netrin-1

For immunohistochemistry, paraffin-embedded sections were rehydrated and treated with 3% hydrogen peroxide for 5–10 min. Slides were blocked in 10% normal goat serum in PBS for 1 h and then incubated with Netrin-1 antibody (1∶250) at 4°C overnight. The Elite Vectastain ABC kit (Vector Laboratories, Burlingame, CA, USA) and Vector DAB substrate were used for detection following the manufacturer's instructions.

### Statistical analysis

Data were expressed as the mean±SD. In all figures, vertical error bars denote the SD. The significance of differences among groups was evaluated by one-way analysis of variance (ANOVA). Tukey and/or LSD method were used as a *post hoc* test for comparison among different groups. *P* values less than 0.05 were considered statistically significant.

## Results

### Expression of netrin-1 in the pancreas during L-Arginine-induced AP

**Figure 1:**
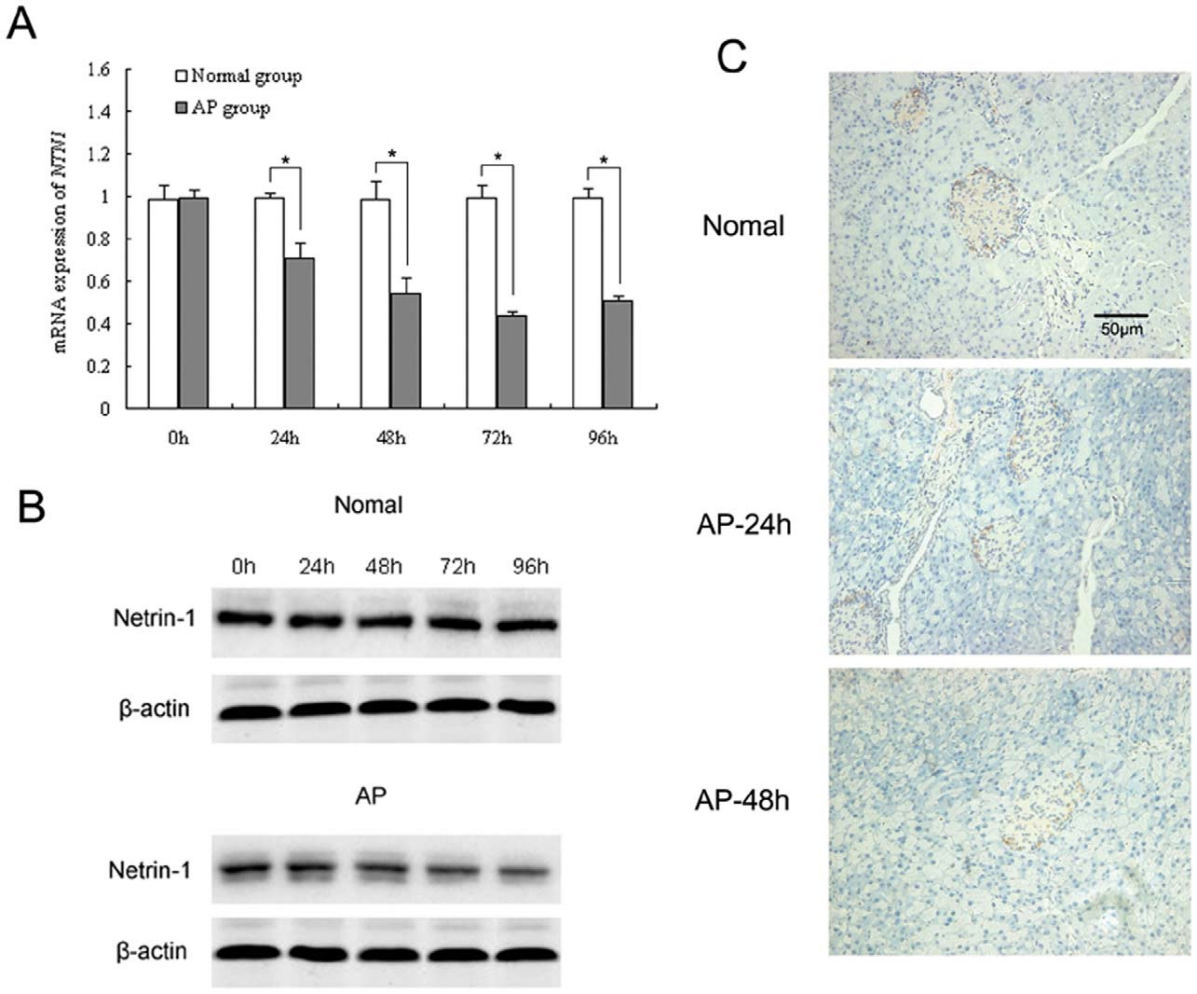
Expression of netrin-1 in the pancreas. RT-PCR (A) and Western blot analysis (B) of netrin-1 expression and immunohistochemical staining (C) of netrin-1 at the indicated times in mouse (C57BL/6) in normal and AP model pancreas. Acute pancreatitis was induced by two intraperitoneal injections of L-Arginine (4 g/kg, 1 h apart). Data are presented as mean±SD (n = 6 per group in normal mice, n = 8 per group in the AP model). ^*^ *P*<0.05.

Expression of netrin-1 in the pancreas was analyzed by RT-PCR and Western blot analysis. The results showed that both netrin-1 mRNA and protein were expressed in the pancreas of the normal group, and both significantly decreased at 24, 48, 72, and 96 h after the AP model was induced compared to the normal group (*P<0.05*, Figure 1A, 1B). Immunohistochemistry showed that in the normal and AP groups, netrin-1 immunoreactivity was mainly seen in islets, and was nearly undetectable in exocrine cells (Figure 1C).

### Effect of netrin-1 on plasma amylase levels in L-Arginine-induced AP

**Figure 2:**
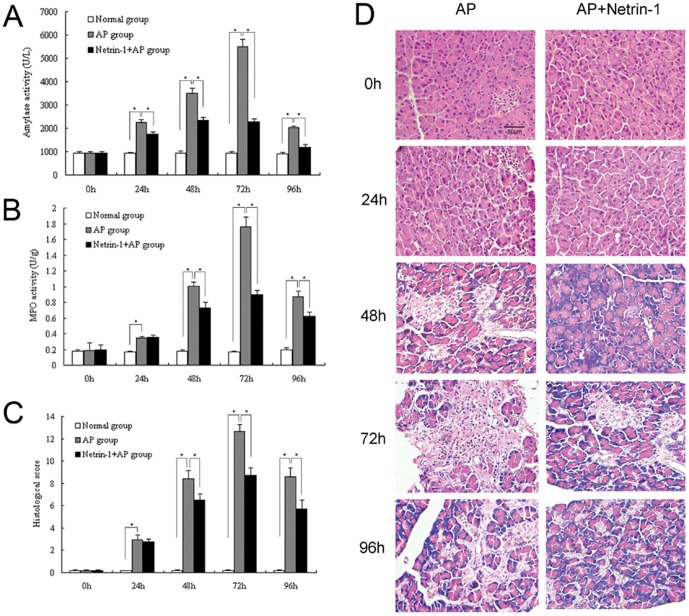
Effect of netrin-1 on pancreatic inflammation. A. Plasma amylase activity. B. Pancreatic myeloperoxidase (MPO) activity. C. Histological scores (sum of interstitial edema, inflammatory cell infiltration, hemorrhage, and acinar cell necrosis). D. Histology. Representative micrographs of H&E-stained pancreatic sections at the indicated times are shown. Acute pancreatitis was induced by 2-hourly two intraperitoneal injections of L-Arginine (4 g/kg, 1 h apart). Mice were treated with recombinant mouse netrin-1 at a dose of 1 µg/mouse or vehicle (0.1% BSA) immediately after the second injection of L-Arginine and every 24 h thereafter. Data are presented as mean±SD (n = 6 per group in the normal mice, n = 8 per group in both the AP model and netrin-1 treated AP model). ^*^ *P*<0.05.

Elevated plasma amylase is an important marker of pancreatic acinar cell injury. Amylase activities markedly increased at 24, 48, 72, and 96 h after the model was induced in the AP group compared to the normal control group (*P<0.05*, Figure 2A). Netrin-1 administration significantly decreased plasma amylase levels compared to the untreated AP group at 24, 48, 72, and 96 h (*P<0.05*, Figure 2A).

### Effect of netrin-1 on pancreas MPO and histology in L-Arginine-induced AP

Pancreatic inflammation was assessed by measuring pancreatic MPO activity and histology. Development of acute pancreatitis is accompanied by sequestration of neutrophils in the pancreas. Measurement of MPO activity in tissue has been used as a biochemical marker of neutrophil infiltration. Pancreatic MPO activity significantly increased upon development of acute pancreatitis, and peaked at 72 h after the AP model was induced, consistent with the time point at which plasma amylase peaked (*P<0.05*, Figure 2B). Netrin-1 administration significantly decreased pancreatic MPO activity at 48, 72, and 96 h after the model was induced compared to the untreated AP group (*P<0.05*, Figure 2B). Histological evaluation of H&E-stained sections of the pancreas revealed tissue damage over time after induction of pancreatitis; however, no histological changes were observed in the normal group. At 0 hour, the histological features of the pancreas in both AP and netrin-1 treated AP groups were typical of a normal architecture. L-Arginine administration resulted in severe AP, as characterized by pancreatic interstitial edema, neutrophil infiltration, hemorrhage, and acinar cell necrosis in the pancreas, with peak injury occurring after 72 h, and without any morphological changes in the Langerhans islets. The presence of edema, neutrophil infiltration, hemorrhage, necrosis, and the total histological scores of pancreas, were significantly reduced in the netrin-1-treated AP group compared to the untreated group after 48, 72, and 96 h (*P<0.05*, Figure 2C, 2D).

### Effect of netrin-1 on lung MPO and histology in L-Arginine-induced AP

**Figure 3:**
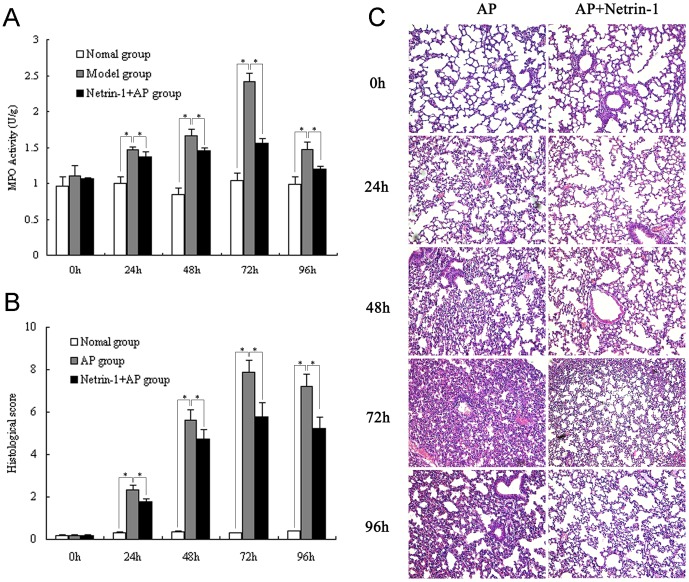
Effect of netrin-1 on lung MPO and histology. A. Lung myeloperoxidase (MPO) activity. B. Histological scores (sum of inflammatory cell infiltration, pulmonary edema, and hemorrhage). C. Histology. Representative micrographs of H&E-stained lung sections at the indicated times are shown. Acute pancreatitis was induced by two intraperitoneal injections of L-Arginine (4 g/kg, 1 h apart). Mice were treated with recombinant mouse netrin-1 at a dose of 1 µg/mouse or vehicle (0.1% BSA) immediately after the second injection of L-Arginine and every 24 h thereafter. Data are presented as mean±SD (n = 6 per group in the normal mice, n = 8 per group in both the AP model and netrin-1 treated AP model). ^*^P<0.05.

Consistent with previous studies, AP induced by two intraperitoneal injections of L-Arginine was associated with lung injury. As shown in Fig. 3A, lung MPO activity was also significantly increased upon development of acute pancreatitis, and peaked at 72 h after the AP model was induced. Netrin-1 administration significantly decreased lung MPO activity at 24, 48, 72, and 96 h after the model was induced compared to the untreated AP group (*P<0.05*). Histological examination of lung tissue sections further confirmed lung injury in L-Arginine-induced AP animals, with the evidence of inflammatory cell infiltration (predominantly neutrophils), pulmonary edema, and hemorrhage of alveoli and interstitial tissue. The most severe injury occurred at 72 h after AP induction. The presence of neutrophil infiltration, pulmonary edema, and hemorrhage, and lung total histological scores, were significantly reduced in the netrin-1-treated AP group compared to the untreated group at the time points of 24, 48, 72, and 96 h (*P<0.05*, Figure 3B, 3C).

### Effect of Netrin-1 on plasma cytokines level in L-Arginine-induced AP

**Figure 4:**
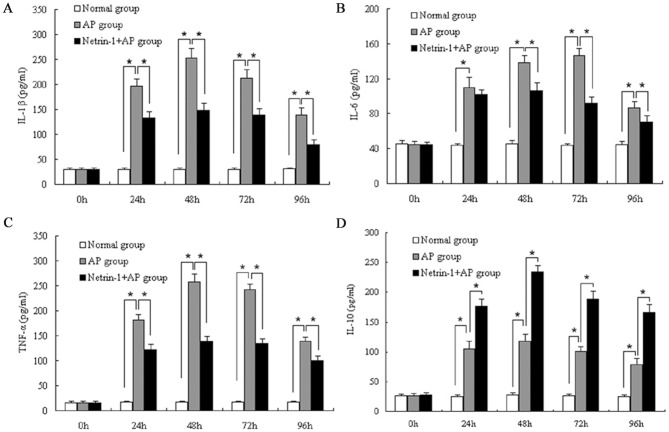
Effect of Netrin-1 on plasma cytokines level. IL-1β, IL-6, TNF-α, and IL-10 plasma concentrations at the indicated times are shown for the normal group and AP groups with and without netrin-1 treatment. Acute pancreatitis was induced by two intraperitoneal injections of L-Arginine (4 g/kg, 1 h apart). Mice were treated with recombinant mouse netrin-1 at a dose of 1 µg/mouse or vehicle (0.1% BSA) immediately after the second injection of L-Arginine and every 24 h thereafter. Data are presented as mean±SD (n = 6 per group in the normal mice, n = 8 per group both in the AP model and netrin-1 treated AP model). ^*^ *P*<0.05.

All measured plasma inflammatory cytokines in the AP group, including IL-1β, IL-6, TNF-α, and IL-10, were significantly higher than that in the normal group at 24, 48, 72, and 96 h after the model was induced (*P<0.05*, Figure 4). Netrin-1 administration significantly decreased the plasma levels of IL-1β, IL-6, and TNF-α, and increased plasma IL-10 levels at several specific time points after the model was induced compared to the untreated AP group (*P<0.05*, Figure 4).

### Effect of netrin-1 on pancreatic NF-κB activation in L-Arginine-induced AP

**Figure 5:**
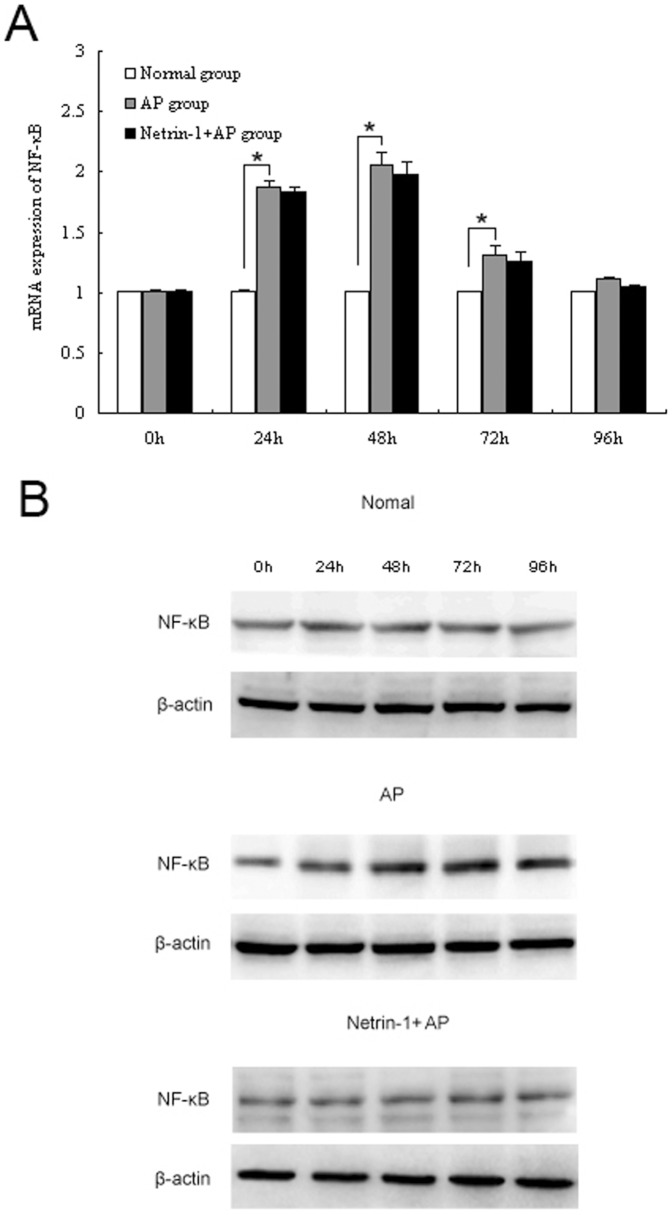
Effect of netrin-1 on pancreatic NF-κB activation. RT-PCR (A) and Western blot analysis (B) of NF-κB p65 expression at the indicated times are shown for the normal group and AP groups with and without netrin-1 treatment. Acute pancreatitis was induced by two intraperitoneal injections of L-Arginine (4 g/kg, 1 h apart). Mice were treated with recombinant mouse netrin-1 at a dose of 1 µg/mouse or vehicle (0.1% BSA) immediately after the second injection of L-arginine and every 24 h thereafter. Data are presented as mean±SD (n = 6 per group in the normal mice, n = 8 per group both in the AP model and netrin-1 treated AP model). ^*^ *P*<0.05.

Expression of NF-κB p65 in the pancreas was analyzed by RT-PCR and Western blot analysis. Both NF-κB p65 mRNA and protein levels in the AP group were significantly increased at 24, 48, and 72 hours after the model was induced compared to the normal group (*P<0.05*, Figure 5A, 5B). There was non-significant down-regulation of NF-κB mRNA and protein expression in the netrin-1-treated AP group compared to the untreated AP group during the course of AP (*P>0.05*, Figure 5A, 5B).

## Discussion

Acute pancreatitis is a life-threatening disease with an unpredictable clinical course that, to date, has no satisfactory therapy [2]–[4]. In the present study, we showed that netrin-1 protects against acute pancreatitis as shown by reductions in plasma amylase, MPO activity, pancreatic and pulmonary tissue damage, and pro-inflammatory cytokine production. To the best of our knowledge, this is the first study to demonstrate a protective effect of netrin-1 on L-Arginin-induced AP.

Netrin-1 has been ascribed a role in pancreatic development, but was previously undetected in adult islet cells in the absence of injury [15]. Yang et al. recently reported that all netrin genes are expressed in adult mouse islets, and netrin receptors are also found in adult mouse and human islets [17]. Consistent with their observations, our immunohistochemical results demonstrated that in both the normal pancreas and L-Arginin-induced AP pancreas, netrin-1 was mainly expressed in the islet cells, and the expression of pancreatic netrin-1 mRNA and protein was down-regulated during the course of AP. However, the mechanism underlying the down-regulation of netrin-1 in AP needs to be further investigated.

Infiltration of leukocytes is one of the hallmarks of tissue injury in acute pancreatitis. Infiltrating leukocytes, mainly composed of neutrophils, contribute to the pathogenesis of acute pancreatitis by producing various pro-inflammatory cytokines and releasing proteases [20], [21]. One potential therapeutic approach for acute pancreatitis is to manipulate the activation and recruitment of neutrophils, and this strategy is supported by observations that depletion of neutrophils attenuated tissue injuries observed in acute murine pancreatitis models [22], [23]. Recent *in vivo* and *in vitro* findings indicate that netrin-1 is a potent inhibitor of leukocyte migration across the endothelium, and may thereby control inflammation [9]. Furthermore, netrin-1 has been shown to modulate inflammatory responses and reduce local tissue injury in several animal models, including kidney ischemia reperfusion injury, hypoxia-induced inflammation, acute lung injury, peritonitis, and inflammatory bowel disease [10]–[14]. To date, the role of netrin-1 in acute pancreatitis has not been reported, however, we postulated that netrin-1 may have beneficial effects in this disease. The method of netrin-1 administration in the present study was selected based on: 1) To look at the prevention or treatment effect of the intervention on pancreatitis, the 3 intervention time points reported in most experimental pancreatitis studies are 1∼6 hours before induction, immediately after the induction, and 1∼6 hours after the induction. 2) In the above literature, studies on the role of netrin-1 in protecting kidney, lung, colon, or peritoneum from inflammatory injury have found netrin-1 can effectively protect these organs/tissues if it is administrated at 2 hours before or immediately after the induction and once every 24 hours thereafter at the dosage of 1 µg/mouse. The present study showed that our netrin-1 administration has protective and beneficial effects on the pancreas and lung in L-Arginin-induced pancreatitis model, as measured both biochemically and histologically.

As expected, acute pancreatitis was successfully and consistently induced in C57BL/6 mice following the administration of two doses of L-Arginin, 4 g/kg each, and 1 h apart. Both plasma amylase and MPO levels in pancreas and lung significantly increased at 24 h after induction of pancreatitis compared to levels in normal animals. Histopathological changes observed in pancreatic acinar cells concurred with the time point at which both plasma amylase and MPO activities started to increase. L-Arginine administration resulted in significant inflammatory cells infiltration and tissue damage in both pancreas and lung. The infiltration of inflammatory cells, with the majority being neutrophils, was also observed at 24 h after L-arginine administration, and was most prominent after 72 h in both pancreas and lung. Netrin-1 treatment significantly reduced neutrophil infiltration both in pancreas and lung as evidenced by MPO levels and histological manifestations compared to untreated animals. Our study shows that netrin-1 treatment significantly ameliorates the severity of L-Arginin-induced AP and the protective effect of netrin-1 against AP was positively related to its ability to inhibit leukocyte infiltration.

Accumulating evidence has shown that pro-inflammatory cytokines, especially TNF-α, IL-1ß, and IL-6, are pivotal in explaining why local pancreatic damage evolves to a systemic disease [24], [25]. In experimental pancreatitis, the plasma levels of TNF-α, IL-1ß, and IL-6 are elevated and their blockade attenuates the disease process [26]–[28]. In the present study, plasma levels of TNF-α, IL-1ß, IL-6, and IL-10 gradually increased after induction of pancreatitis, with peak levels occurred after 48 or 72 h. We observed that netrin-1 treatment resulted in significant reduction in plasma levels of TNF-α, IL- 1ß, and IL-6. Furthermore, netrin-1 treatment led to a significant increase in plasma IL-10 levels. Our results demonstrate that Netrin-1 can regulate inflammatory cytokines and suppress the pro-inflammatory response in AP.

The signaling pathway responsible for the role of netrin-1 in regulating leukocyte infiltration and cytokine production during the course of AP has been of interest. One important signaling molecule, NF-κB, was identified as an important regulator of the expression of many inflammatory mediators in the pancreas [29]. NF-κB is a nuclear transcription factor responsible for regulating the transcription of a wide variety of genes involved in immunity and inflammation. There is an emerging body of evidence which suggests that NF-κB plays an important role in the early stage of acute pancreatitis, and that inhibiting this transcription factor reduces the disease severity [19], [30]–[32]. Although most researchers agree that blocking NF-κB activation is beneficial in acute experimental pancreatitis, the opposite effect was seen in other studies investigating cerulein-induced pancreatitis [33], [34]. Our colleagues recently demonstrated that hydrogen-rich saline treatment can markedly alleviate the NF-κB activation and ameliorate disease severity in a rat model of L-Arginin-induced pancreatitis [35]. To date, there is limited data on NF-κB activation in the mouse model of L-Arginin-induced pancreatitis. Here, we show that NF-κB activation gradually increased after induction of pancreatitis, and positively correlated with an increase in plasma pro-inflammatory cytokines, as well as plasma amylase and the influx of inflammatory cells into the pancreas. It is remarkable that recent research has shown NF-κB activation is involved in the regulation of netrin-1 expression in the context of inflammation. Pradisi et al. demonstrated in a mouse model of inflammation induced colorectal cancer, the netrin-1 gene is a direct transcriptional target of NF-κB and that NF-κB activation triggers the up-regulation of netrin-1 [36]. Consistent with their findings, Aherne et al. also reported that netrin-1 is induced by inflammatory signaling pathways in intestinal epithelia via NF-κB signaling during acute experimental colitis [14]. Other authors, however contrary to the above two reports, have reported that netrin-1 is repressed during the acute inflammatory response [9], [12], [13]. Mirakaj et al. showed that netrin-1 was repressed during periods of inflammatory lung injury *in vivo*, and they demonstrated the involvement of a NF-κB–dependent mechanism in the transcriptional repression of netrin-1 [12]. In line with these observations, our data showed that NF-κB activation may be responsible for the down-regulation of pancreatic netrin-1 expression in L-Arginin-induced pancreatitis. Furthermore, our RT-PCR and Western blot analyses showed that netrin-1 administration did not significantly inhibit the pancreatic expression of NF-κB in L-Arginin-induced pancreatitis. Biological functions of NF-κB could be more accurately determined by NF-κB DNA binding assay or NF-κB dependent luciferase reporter assay [37], [38]. To further clarify the role of netrin-1 on the function of NF-κB in L-Arginin-induced AP, the NF-κB function should be accurately determined in addition to the expression levels. It is more noticeable that a number of downstream signaling pathways have been investigated in netrin-1-mediated tissue protection [12]–[14], [39]–[43]. The transmembrane receptors, which are deleted in colorectal cancer, the uncoordinated receptor (mouse UNC5A-D; human UNC5H1-4), and the A2B adenosine receptor represent the known netrin-1 receptors. Independent studies have demonstrated that either the UNC5B or A2B adenosine receptor signaling pathway is responsible for netrin-1 inhibition of leukocyte recruitment and the inflammatory response [12]–[14], [43]. With certain limitations, the netrin-1 receptor signaling pathway has not been further investigated in our present study. Based on what we have found, however, we believe that the netrin-1 dependence receptor-mediated signal transduction could be an important mechanism for the protective effect of netrin-1 on AP. However, for netrin-1 therapeutic utility, cautions should be taken as induction of netrin-1 expression via NF-κB in inflammatory bowel disease has been reported to affect colorectal tumor promotion and progression, and is also associated with worse outcome in poorly differentiated pancreatic adenocarcinomas [44], [45]. Therefore, to further study netrin-1 receptor signaling pathway is of critical significances, as it may provide important clues for identifying novel treatment targets for pancreatitis and even pancreatic cancer.

In conclusion, our present study demonstrates for the first time that netrin-1 is capable of improving pancreatic and pulmonary injury and exerting anti-inflammatory effects by inhibiting leukocyte infiltration in mice with severe acute pancreatitis. Therefore, netrin-1 may constitute a novel target in the management of AP.
